# METTL3 Contributes to Osteosarcoma Progression by Increasing DANCR mRNA Stability *via* m6A Modification

**DOI:** 10.3389/fcell.2021.784719

**Published:** 2022-01-12

**Authors:** Xinying Zhou, Yang Yang, Yuejun Li, Guojun Liang, Dawei Kang, Bing Zhou, Qingchu Li

**Affiliations:** ^1^ Department of Spine Surgery, Center for Orthopaedic Surgery, The Third Affiliated Hospital of Southern Medical University, Guangzhou, China; ^2^ Department of Orthopedics, Longtan Hospital of Guangxi Autonomous Region, Liuzhou, China

**Keywords:** osteosarcoma, m6A modification, METTL3, lncRNA DANCR, mRNA stability

## Abstract

**Background:** Osteosarcoma (OS) is the most prevalent bone cancer among children and adolescents, with relatively high mortality rates. RNA N6-methyladenosine (m6A) is the most common human mRNA modification with diverse functions in a variety of biological processes. Previous studies indicated that methyltransferase-like 3 (METTL3), the first methyltransferase to be identified, acted as an oncogene or tumor suppressor in multiple human cancers. However, its functions and underlying mechanisms in OS progression remain unclear; therefore, we explored these processes.

**Methods:** We used real-time quantitative PCR (RT-qPCR) and Western blot assays to explore METTL3 expression in OS tumor tissues and five OS cell lines to assess its clinical significance. To further examine the functional role of METTL3 during OS progression, CCK-8 analyses, transwell assays, and xenograft model studies were conducted after silencing METTL3. Additionally, underlying mechanisms were also explored using RIP-seq and RIP-qPCR approaches.

**Results:** METTL3 was upregulated in OS tumor tissues and cell lines and was associated with a worse prognosis. Moreover, METTL3 silencing suppressed OS cell proliferation, migration, and invasion. Also, *in vivo* METTL3 oncogenic functions were confirmed in the **x**enograft model. Comprehensive mechanistic analyses identified long non-coding RNA (lncRNA) DANCR as a potential target of METTL3, as indicated by reduced DANCR levels after METTL3 silencing. Also, lncRNA DANCR knockdown repressed OS cell proliferation, migration, and invasion. Furthermore, both METTL3 and lncRNA DANCR silencing significantly suppressed OS growth and metastasis. Finally, we hypothesized that METTL3 regulated DANCR expression *via* m6A modification-mediated DANCR mRNA stability.

**Conclusion:** METTL3 contributes to OS progression by increasing DANCR mRNA stability *via* m6A modification, meaning that METTL3 may be a promising therapeutic target for OS treatment.

## Introduction

Osteosarcoma (OS) is the most common primary malignant bone tumor and is characterized by the formation of immature bone or osteoid by cancer cells. The disease usually develops in children and adolescents, thus ranking it as the fifth most frequent cancer among 15–19-year-olds (Durfee et al., 2016; De Azevedo et al., 2020). Although a combined therapy approach of surgical tumor removal and chemotherapy has increased survival rates, the 5-year survival rate of OS patients remains unsatisfactory (Isakoff et al., 2015). Based on previous reports, patients with localized OS have a 5-year survival rate of approximately 70%, whereas the rate of those with metastatic OS varies from 11% to 30% (Harrison et al., 2018). Moreover, approximately 80% of OS patients having undergone surgical treatment may relapse, thus leading to a poor prognosis (Briccoli et al., 2010). Therefore, underlying mechanisms must be identified to generate novel therapeutic strategies for OS treatment.

Over the past decade, epigenetic modifications, including histone modifications, chromatin remodeling, and DNA methylation, have been identified with key roles in tumorigenesis (Flavahan et al., 2017). Notably, N6-methyladenosine (m6A) modification is a biological process that adds or deletes a methyl (CH_3_) group to/from the nitrogen six position of the adenine nucleotide. This is one of the most prevalent RNA modifications and appears to exert many functions in multiple processes *via* RNA translation, pre-mRNA splicing, and RNA stability (Dominissini et al., 2012; Deng et al., 2018). Collectively, a couple of modulators, including methylases (writers) and demethylases (erasers), are responsible for m^6^A modification (Liu et al., 2014; Ping et al., 2014). Also, m^6^A-modified RNA may be recognized by m^6^A-binding proteins (readers) (Xiao et al., 2016; Shi et al., 2017). Moreover, previous studies reported the regulatory role of m^6^A modification in several malignant tumors (including OS) (Deng et al., 2018; Sun et al., 2019). For example, methyltransferase-like 3 (METTL3), the first methylase to be identified, was dysregulated in multiple malignant tumor types, including colorectal cancer (Peng et al., 2019), bladder cancer (Han et al., 2019), lung cancer (Lin et al., 2016), and melanoma (Dahal et al., 2019). Lin et al. reported that the oncogenic role of METTL3 in lung cancer, as confirmed by loss- and gain-of-function studies, was mediated by promoting the translation of oncogenes *via* interactions with translation initiation machinery (Lin et al., 2016). Also, METTL3 was reportedly upregulated in breast cancer cells and tissues, with gene silencing significantly inhibiting cell proliferation and promoting apoptosis by regulating Bcl-2, thereby indicating an oncogenic role for METTL3 in breast cancer progression (Wang et al., 2020). However, few studies have reported METTL3 functions during OS progression.

Long non-coding RNAs (lncRNAs) are transcripts that exceed 200 nucleotides, with no protein coding abilities, but with vital roles in diverse biological processes and disease etiology (including tumorigenesis) (Fang and Fullwood, 2016). Among these molecules, lncRNA DANCR is a newly identified oncogene implicated in diverse malignances, including gastric cancer (Pan et al., 2018), breast cancer (Tang et al., 2018), and hepatocellular carcinoma (Ma et al., 2016), and may function as a competing endogenous RNA or be involved in the epigenetic regulation/modulation of gene expression.

We investigated the potential functions and mechanisms of m^6^A modifications underlying OS progression. Our remit was to characterize the role of METTL3 and its associated mechanisms. Our preliminary data suggested that METTL3 contributed to OS progression by increasing DANCR mRNA stability *via* m6A modification.

## Materials and Methods

### Tissue Samples

Forty pairs of human OS tissues and corresponding adjacent tissues were collected at The Third Affiliated Hospital of Southern Medical University from July 2013 to July 2015 and stored at −80°C until required. No patients had chemotherapy before surgery. Written informed consent was obtained from all patients. The study was approved by the ethics committee of our university.

### Cell Culture and Transfection

Five OS cell lines, Saos-2, SJSA-1, MG63, HOS, and U-2 OS, and the human osteoblast cell line, human fetal osteoblastic (hFOB) 1.19 cells, were obtained from the American Type Culture Collection (ATCC). Cells were cultured in DMEM medium (Cat# SH30243.01, HyClone™, Cytiva, Marlborough, MA, USA), RPMI medium 1,640 (Cat# 11875093, Thermo Fisher Scientific, Waltham, MA, USA), and DMEM/F12 medium (Cat# D9785, Sigma). All media were supplemented with 10% fetal bovine serum (FBS) (Cat# 04-001-1ACS; BI) and 1% penicillin/streptomycin (Cat# C0222, Beyotime, Shanghai, China). OS cell lines were maintained at 37°C in 5% CO_2_, whereas hFOB 1.19 cells were cultured under 3°C.

The small interfering RNA (si-RNA) oligonucleotide targeting lncRNA DANCR and the matched negative control were generated by GenePharma (Shanghai, China). Lipofectamine 2000 (Cat# 11668019; Thermo Fisher Scientific) for transfections was used according to the manufacturer’s protocols.

### CRISPR-Cas9 Knockout of METTL3

METTL3 deletion was performed using CRISPR/Cas9 technology. HOS and U-2 OS cells were transfected with METTL3 CRISPR/Cas9 and homology-directed repair (HDR) plasmids. After this, stable cells were collected.

### Cell Counting Kit-8 Assay

OS cell line proliferation was determined by Cell Counting Kit-8 (CCK8) assay. Approximately 2 × 10^4^ cells were added to wells in 96-well plates. After 24, 48, 72, and 96 h of growth, a CCK-8 working solution (Cat# ab228554, Abcam, Cambridge, MA, USA) was added to plates, followed by further incubation for 1–4 h. The OD at 450 nm was then recorded on a microplate reader.

### RT-qPCR

Total RNA from cells and tissues was extracted using TRIzol reagent (Invitrogen, Carlsbad, CA, USA) following the manufacturer’s protocols and cDNA synthesis performed using the HiScript III first-strand cDNA synthesis kit (Cat# R312-01, Vazyme, Nanjing, China). Then, PowerUp™ SYBR^®^ Green instrumentation (Cat# A25742; Thermo Fisher) was used to detect mRNA expression levels. GAPDH and U6 were used for expression normalization. Primers for RT-qPCR are listed (Table S1).

### Western Blotting

Human OS tissue and cell lines were lysed in RIPA lysis buffer (Cat# P0013B, Beyotime) supplemented with a 1× protease inhibitor cocktail (Cat# P1005, Beyotime) for 30 min at 4°C, followed by a 15-min centrifugation step at 12,000 × *g* to generate supernatants. Then, a bicinchoninic acid protein assay kit (Cat# ab102536, Abcam) was used to determine protein concentrations. Next, 5× protein loading buffer (Cat# P0015; Beyotime) was added to lysates and denatured for 5 min at 100°C. Approximately 20 μg protein was separated using sodium dodecyl sulfate–polyacrylamide gel electrophoresis and electrophoretically transferred to polyvinylidene fluoride membranes. Membranes were then blocked in 5% fat-free milk for 45 min at room temperature, followed by an overnight incubation with primary antibodies at 4°C. Next, membranes were rinsed three times in 1× TBST buffer and further incubated with horseradish peroxidase (HRP)-conjugated secondary antibodies for 60 min at room temperature. Protein signals were determined using enhanced chemiluminescence reagent (Cat# K-12045-D10, Advansta, San Jose, CA, USA). Antibodies were rabbit anti-Mettl3 (Cat# 15073-1-AP, Proteintech, Wuhan, China), rabbit anti-*β*-actin (1:3,000, Cat# 4,970, Cell Signaling, Danvers, MA, USA), and goat anti-rabbit IgG H&L (HRP) (1:1,500, Abcam, Cat# ab205718).

### Migration and Invasion Assays

In the first day, both two OS cell lines were transfected, followed by trypsinization and resuspension in serum-free medium. Cells were then added to transwell chambers at a density of 3×10^4^ cells. After a 24-h culture, chambers were carefully removed from wells and a cotton-tipped bud was used to wipe cells in the upper chamber. Then, after 20 min of fixation in 4% paraformaldehyde, 0.1% crystal violet was added to the upper chamber to stain cells for approximately 20 min. Finally, cells were rinsed in 1× phosphate-buffered saline (PBS) to remove excess crystal violet. Migrated cell numbers were calculated using an inverted microscope. For invasion assays, protocols were similar to migration assays except chamber inserts were coated with Matrigel.

### RNA Immunoprecipitation qPCR

The RIP assay was performed using a previously described method (Gagliardi and Matarazzo, 2016). Briefly, OS cells were lysed and approximately 1%–2% of the lysate used as the input. Then, supernatants were incubated with 5 μg of the indicated antibody for crossed-linking with A/G magnetic beads (Cat# 17-10085/86, Millipore) overnight at 4°C, including normal rabbit IgG (Cat# AC005, ABclonal, Woburn, MA, USA), anti-METTL3 (Cat# A301-567A, Bethyl Laboratories, Montgomery, TX, USA), anti-METTL14 (Cat# ab98166, Abcam), anti-YTHDF1 (Cat# 86,463, Cell Signaling), anti-YTHDF2 (Cat# 24744-1-AP, Proteintech), anti-YTHDF3 (Cat# ab103328, Abcam), anti-WATP (Cat# 56,501, Cell Signaling), anti-FTO (Cat# 14,386, Cell Signaling), and anti-ALKBH5 (Cat# ABE547, Millipore, Bedford, MA, USA). The next day, excess antibodies were washed five times in RIPA buffer. TRIzol reagent was used to collect input and RNA samples, which were subjected to RT-qPCR analysis. Relative enrichment was determined by calculating the cycle threshold values of RIP samples relative to input samples.

### Animal Studies

For *in vivo* studies, BALB/c-nu/nu mice (male, body weight = 18–22 g, and 6 weeks old) were purchased from the Shanghai Research Center of the Southern Model Organisms. All experimental protocols were performed under the guidelines of the Animal Care and Use Committee of The Third Affiliated Hospital of Southern Medical University. Xenograft tumors were established by subcutaneous injection with stable cell lines (3,000,000 per point).

### Statistical analysis

Data were processed in GraphPad Prism (version eight; GraphPad Inc, La Jolla, San Diego, CA, USA) and represented as the mean ± standard error of the mean (SEM). The log-rank test was used to calculate patient survival associated with low or high METTL3 levels. The statistical significance of METTL3 levels in OS tissues or para-tissues was determined using paired Student t tests. Correlations between METTL3 and DANCR were calculated using Spearman correlation tests. Student t tests or one-way analysis of variance followed by Tukey’s analyses were used to assess significant differences between different groups. Finally, *p* < 0.05 was considered statistically significant.

## Results

### METTL3 is Upregulated in OS Tumor Tissues and Cell Lines

**FIGURE 1:**
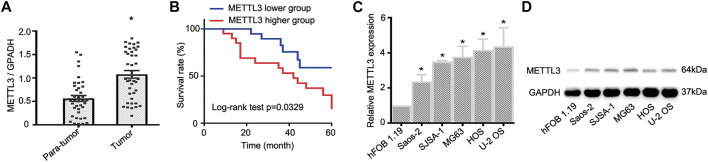
The expression profile of METTL3 in osteosarcoma (OS) tumor tissues and cell lines. **(A)** RT-qPCR analysis of METTL3 mRNA levels in OS tumor tissues and matched normal tissues (n = 40). **(B)** Kaplan–Meier curve analyses indicated that patients with higher METTL3 levels had a relatively poor prognosis. **(C)** RT-qPCR and **(D)** Western blotting showing increased METTL3 levels in Saos-2, SJSA-1, MG63, HOS, and U-2 OS cells when compared with human fetal osteoblastic cells. *p < 0.05.

To document METTL3 function during OS progression, 40 pairs of OS tumor tissues and adjacent normal tissues were used to examine METTL3 expression using RT-qPCR. As shown (Figure 1A), when compared with paired normal tissue, METTL3 was significantly increased in OS tumor samples. Moreover, to identify the clinical significance of METTL3 in OS progression, patients were divided into two groups with reference to mean METTL3 values and comprised a higher group (METTL3 levels >mean values) and a lower group (METTL3 levels <mean values). Accordingly, patients with high METTL3 expression levels exhibited a relatively lower survival rate (Figure 1B). Furthermore, to examine METTL3 profiles in OS cells, five OS cell lines (Saos-2, SJSA-1, MG63, HOS, and U-2 OS) and the human osteoblast cell line, hFOB 1.19, were used. As confirmed by RT-qPCR and Western blotting, METTL3 expression was elevated in all OS cell lines when compared with hFOB 1.19 cells (Figures 1C,D). Taken together, METTL3 may act as an oncogene during OS progression.

### METTL3 Promotes OS Cell Proliferation, Migration, and Invasions

**FIGURE 2:**
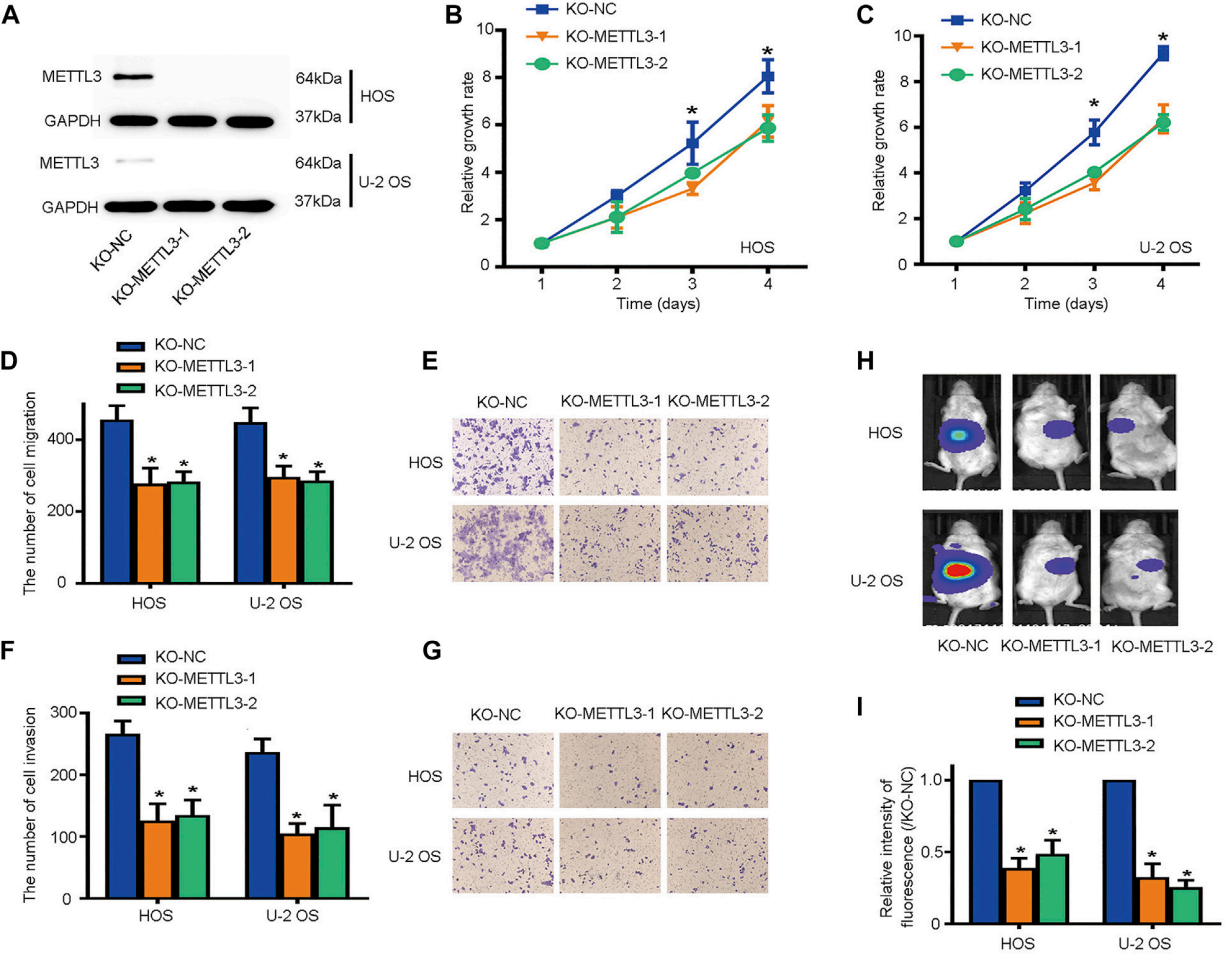
METTL3 silencing suppresses osteosarcoma (OS) cell proliferation, migration, and invasion. **(A)** Western blotting showing METTL3 knockout efficiency in two OS cell lines. Growth curves of HOS **(B)** and U-2 OS **(C)** cells after METTL3 silencing using CCK-8 assays at indicated times. Transwell assays were used to examine the migration abilities of HOS and U-2 OS cells. **(D)** Quantitation of migrating cells passing through membrane without Matrigel. **(E)** Representative images of OS cell migration. Transwell assays were used to examine the invasion activities of HOS and U-2 OS cells. **(F)** Quantitation of migrating cells passing through membranes plus Matrigel. **(G)** Representative images of OS cell invasion. **(H,I)** Tumor volumes curves at indicated time points. *p < 0.05.

To identify other potential METTL3 functions during OS progression, we deleted METTL3 using CRISPR/Cas9 technology; knockout efficiency was confirmed by western blotting (Figure 2A). Then, CCK-8 assays were used to assess cell proliferation. As shown (Figures 2B,C), METTL3 silencing effectively suppressed OS cell proliferation. Moreover, it is accepted that metastasis contributes to the majority of cancer-related deaths. Thus, we used transwell assays to assess the migratory and invasive properties of OS cells after METTL3 knockout. Accordingly, METTL3 silencing reduced OS cell numbers passing through membranes, either with or without Matrigel, suggesting that METTL3 promoted OS cell migration and invasion (Figures 2D–G). To further investigate METTL3 pathological functions in *in vivo* OS progression, BALB/c-nu/nu mice were subcutaneously injected with stable cell lines at indicated times. As shown (Figure 2H), METTL3 knockout cells decreased tumor volumes when compared with tumors in the matched control group. Thus, METTL3 appeared to function as an oncogene by promoting OS cell proliferation, migration, and invasion.

### METTL3-Dependent m6A Modifications Regulate lncRNA DANCR

**FIGURE 3:**
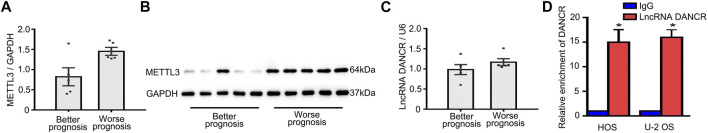
Identification of potential METTL3 targets during OS progression. **(A)** Patient METTL3 mRNA and **(B)** protein levels associated with better and worse prognoses. DANCR expression **(C)** in patients with better and worse prognoses. **(D)** RIP-qPCR showing a relatively higher enrichment of DANCR. *p < 0.05.

To examine mechanisms underlying METTL3-mediated OS progression, five patients with good prognoses, as confirmed by no outcome events with a long follow-up duration, and five patients with the shortest follow-up times were selected. As shown in Figure 3A, patients reporting better prognoses displayed lower METTL3 mRNA levels. Identical to mRNA levels, higher METTL3 protein levels were identified in patients with worse prognoses (Figure 3B). According to previous studies, lncRNA DANCR functioned as an oncogene in several cancers. We then hypothesized whether oncogenic METTL3 activities during OS progression were mediated by DANCR. Interestingly, patients reporting better prognoses exhibited lower DANCR mRNA levels (Figure 3C). Moreover, RIP-qPCR assays showed that DANCR exhibited relatively higher levels of m6A modification when compared with controls (Figure 3D). Thus, DANCR could be a potential target of METTL3 during OS progression.

### LncRNA DANCR Promotes OS Cell Proliferation, Migration, and Invasion

**FIGURE 4:**
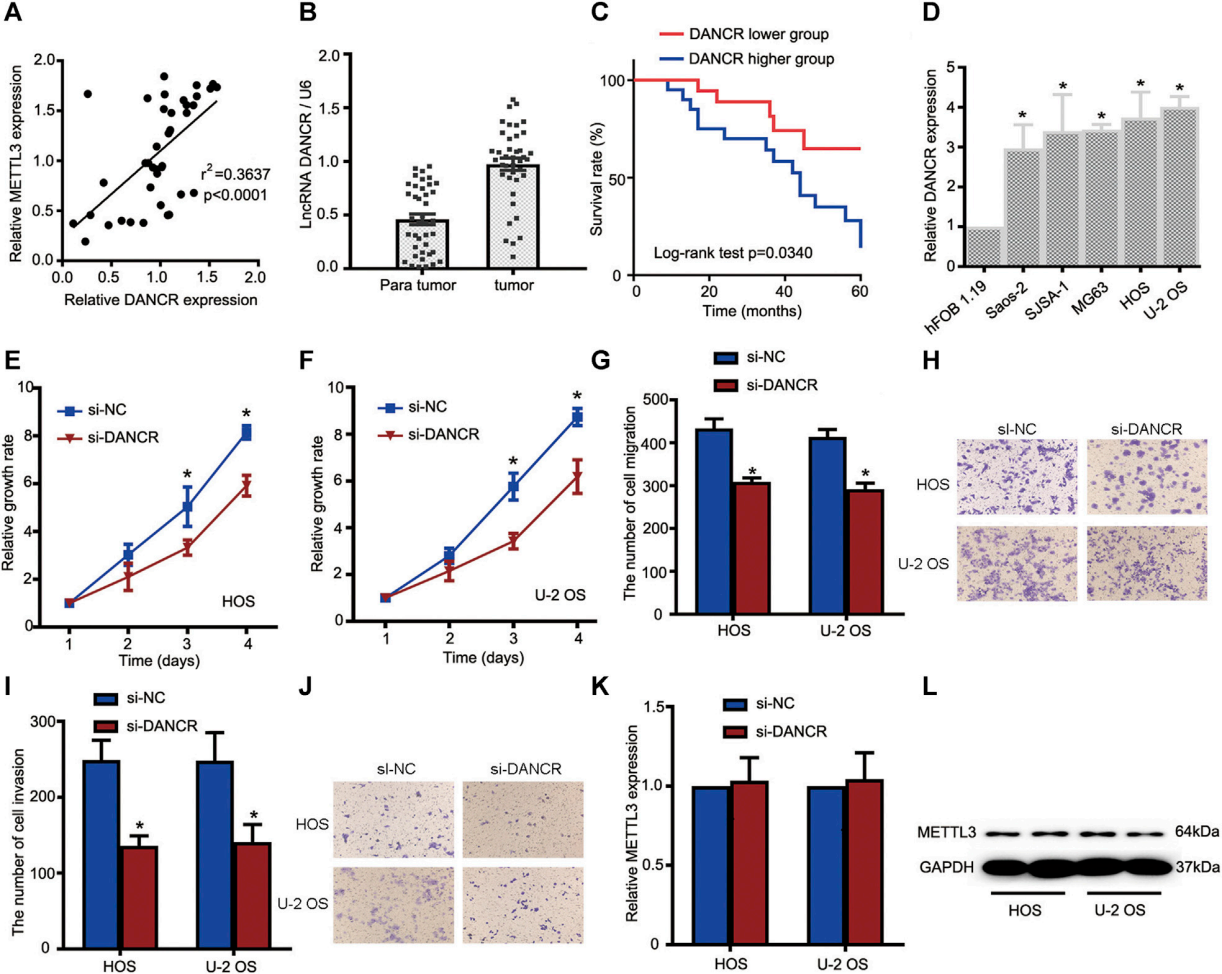
The METTL3/lncRNA DANCR axis promotes OS cell tumorigenesis and progression. **(A)** Correlations between METTL3 and DANCR expression in OS tissue (*n* = 40). **(B)** RT-qPCR showing increased DANCR levels in OS tumor tissues when compared with matched normal tissues. **(C)** Kaplan–Meier survival analyses of patients with OS. Patients with higher DANCR levels showed a relatively poor prognosis. **(D)** RT-qPCR showing elevated DANCR expression in Saos-2, SJSA-1, MG63, HOS, and U-2 OS cells compared with human fetal osteoblastic cells. CCK-8 assays were used to determine viability in HOS **(E)** and U-2 OS **(F)** cells transfected with si-DANCR or the corresponding control. Transwell assays were used to assess the migration activities of HOS and U-2 OS cells. **(G)** Quantitative analyses of migrating cells passing through membranes without Matrigel. **(H)** Representative images of OS cell migration. Transwell assays were used to examine the invasion activities of HOS and U-2 OS cells. **(I)** Quantitative analyses of migrating cells passing through membranes plus Matrigel. **(J)** Representative images of OS cell invasion. **(K,L)** Relative METTL3 expression with or without si-DANCR infected. *p < 0.05.

To gain clear molecular insights into the functional role of DANCR during OS progression, a comprehensive assessment of DANCR, including clinical significance and loss-of-function assays, was performed. First, Spearman correlation tests indicated that DANCR expression was positively correlated with METTL3 expression in OS tissues (Figure 4A). Moreover, elevated DANCR expression was detected in OS tissues when compared with paired normal tissues (Figure 4B). Also, consistent with a previous report, our Kaplan–Meier analyses showed improved survival rates in patients with lower DANCR levels than those with higher levels (Figure 4C).

Based on these clinical data, we investigated DANCR’s role in OS cells. Firstly, DANCR expression was examined in all OS cells and hFOB 1.19 cells. Our RT-qPCR analyses demonstrated significantly increased DANCR levels in OS cells when compared with hFOB 1.19 cells (Figure 4D). We next performed CCK-8 assays to determine cell proliferation after DANCR silencing. As shown in Figures 4E,F, DANCR knockdown significantly reduced HOS and U-2 OS cell proliferation. Also, transwell assays showed that DANCR silencing suppressed HOS and U-2 OS cell migratory and invasive capacities, suggesting an oncogenic role for the METTL3/lncRNA DANCR axis (Figures 4G–J). To further uncover regulatory mechanisms, lncRNA DANCR knockdown was performed to assess METTL3 expression; METTL3 levels were consistent before and after DANCR silencing (Figure 4K–L). Thus, the oncogenic role of METTL3 may have been mediated by DANCR.

### The Oncogenic Role of METTL3 is Mediated by lncRNA DANCR Activation

**FIGURE 5:**
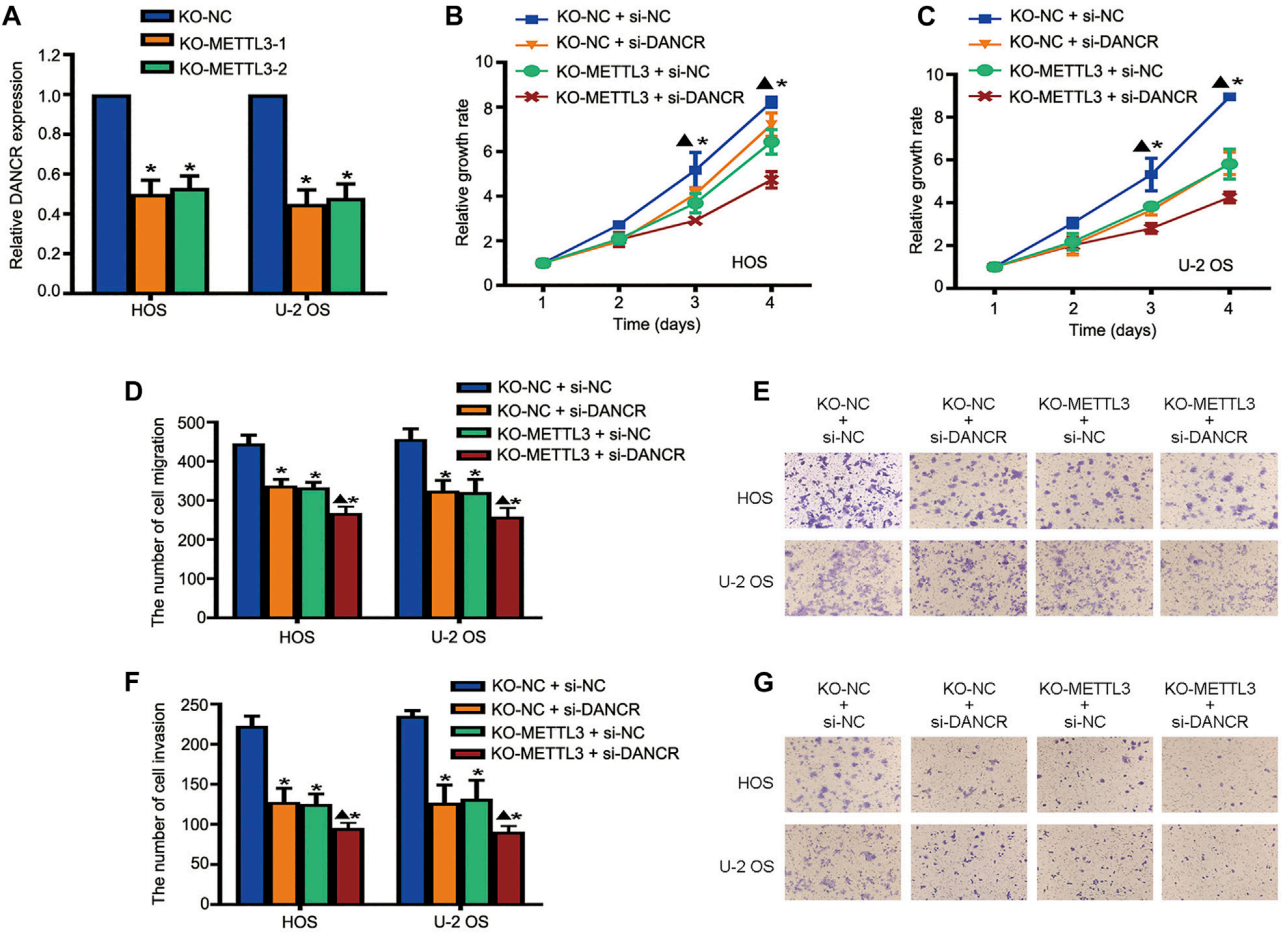
Long noncoding RNA (lncRNA) DANCR is involved in METTL3-mediated tumorigenesis. **(A)** RT-qPCR analysis of DANCR expression after METTL3 silencing. Cell viability of DANCR-silenced, METTL3-silenced, and DANCR and METTL3-silenced HOS cells **(B)** and U-2 OS cells **(C)** at indicated time points. Migration activities of DANCR-silenced, METTL3-silenced, and DANCR and METTL3-silenced HOS and U-2 OS cells. **(D)** Quantitative analyses of migration assay results. **(E)** Representative images of OS cell migration. Invasion activities of DANCR-silenced, METTL3-silenced, and DANCR and METTL3-silenced HOS and U-2 OS cells. **(F)** Quantitative analyses of invasion assay results. **(G)** Representative images of OS cell invasion. *p < 0.05.

To further investigate regulatory mechanisms underlying the oncogenic role of METTL3 during OS progression, DANCR expression after METTL3 silencing was explored. METTL3 silencing suppressed DANCR expression in two OS cell lines (Figure 5A). Moreover, to further confirm that the oncogenic role of METTL3 was mediated by DANCR activation, CRISPR/Cas9-METTL3-silenced cells were transfected with si-DANCR or a corresponding control, followed by CCK-8 assay. Consistent with previous findings, silencing METTL3 or DANCR reduced cell viability. Moreover, after silencing both METTL3 and DANCR, we identified more reduced cell proliferation, suggesting that DANCR may be a potential downstream target of METTL3 (Figures 5B,C). Concordantly, after silencing both METTL3 and DANCR, OS cells passing through membranes, with or without Matrigel, were reduced when compared to individually silencing METTL3 or DANCR (Figures 5D–G).

### METTL3-Mediated m6A Methylation Promotes lncRNA DANCR Stability

**FIGURE 6:**
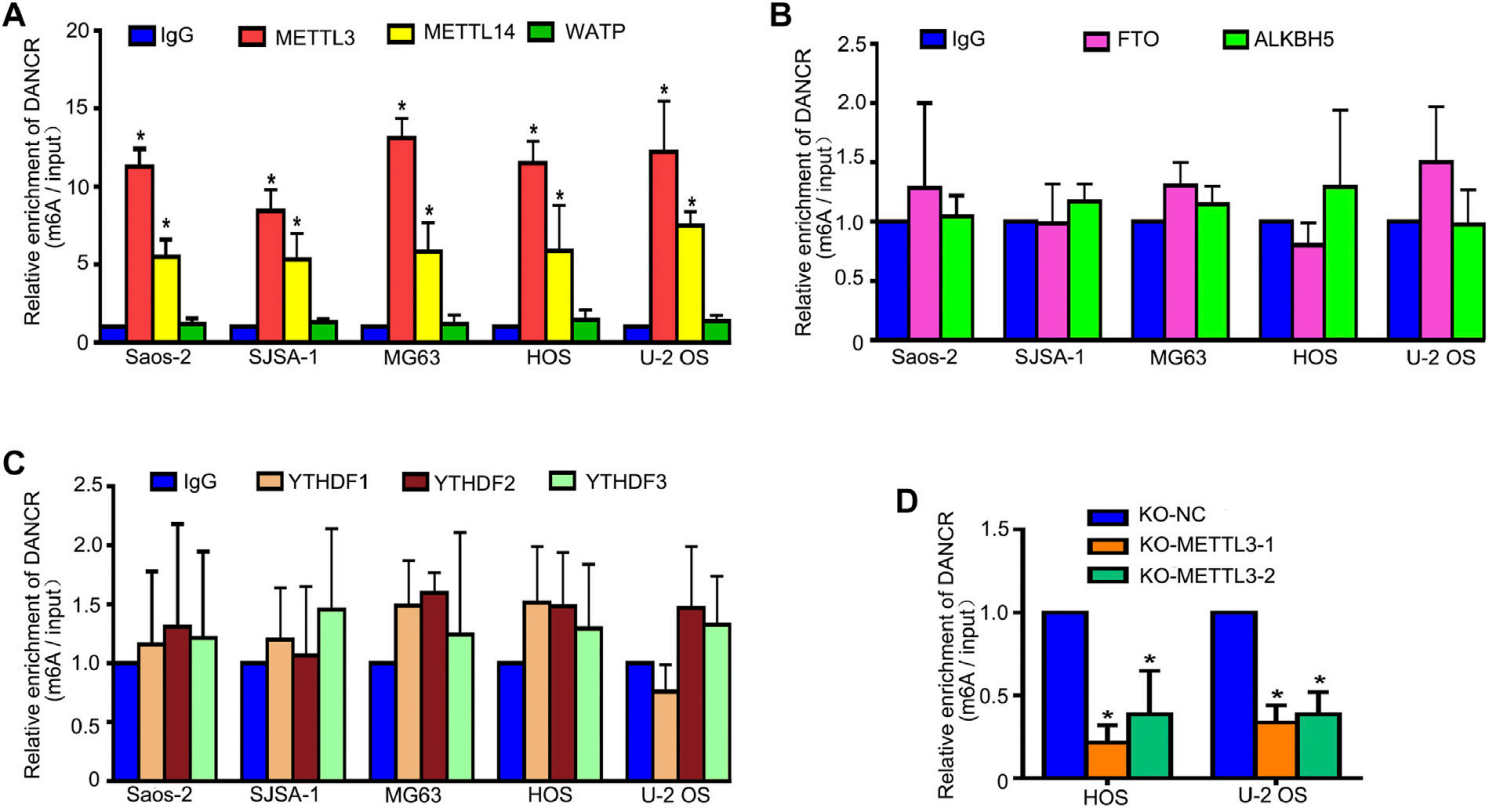
METTL3 increases DANCR mRNA stability *via* m6A modification. We detected interactions between DANCR and METTL3, METTL14, and WTAP **(A)**, FTO and ALKBH5 **(B)**, and YTHDF1, YTHDF2, and YTHDF3 **(C)** by RIP-qPCR assays in five OS cell lines. **(D)** DANCR enrichment by RIP-qPCR assay using the METTL3 antibody.

The dynamic and reversible regulation of m6A modification is regulated by methylases (writers), demethylases (erasers), and m^6^A binding proteins (readers). Representative genes were selected for testing. The methyltransferase complex, which contains METTL3, METTL14, and Wilms tumor 1-associated protein (WTAP), mediates the catalyzation of m6A modification. The two demethylases, fat mass and obesity-associated protein (FTO) and *α*-ketoglutarate-dependent dioxygenase AlkB homolog 5 (ALKBH5), are responsible for removing methylation signatures. Additionally, m6A readers, which function as modulators to recognize m6A-modified targeted RNA, are made up of YTH-domain family (YTHDF)1-3 and promote the translation and degradation of m6A-modified mRNAs. Thus, to explore the METTL3 regulation of DANCR expression, multiple RIP-qPCR assays were performed. As shown in Figures 6A–C, in all five OS cell lines, DANCR enrichment was detected only when RIP assays used METTL3 and METTL14 antibodies. Consistently, METTL3 silencing reduced DANCR enrichment (Figure 6D). Taken together, METTL3 appeared to regulate DANCR RNA stability.

## Discussion

OS is one of the most prevalent cancers among children and adolescents, with a heterogeneous presentation and relatively high mortality rates (Broadhead et al., 2011). Although the overall survival rate has improved due to combinative treatments, many patients still experience poor prognoses (Harrison et al., 2018). Thus, a comprehensive examination of mechanisms driving OS progression is required to identify OS treatments.

As described, m6A modifications are highly prevalent human mRNA modifications and play key roles in cancer progression. Moreover, of the m6A modulators, METTL3 is a key component of the m6A methylation complex and is reportedly involved in hepatocellular carcinoma (Chen et al., 2018), breast cancer (Wang et al., 2020), and colon cancer (Peng et al., 2019). In particular, the oncogenic role of METTL3 in OS progression has been reported by several groups (Miao et al., 2019; Zhou et al., 2020). Zhou et al. showed that METTL3 was upregulated in OS tissue and cells (Zhou et al., 2020) in accordance with our findings. More importantly, high METTL3 expression was also correlated with a poor patient prognosis. Also, we showed that METTL3 silencing suppressed OS cell proliferation, migration, and invasion, suggesting that METTL3 could function as a potential therapeutic target for OS treatment. Our xenograft model further validated the oncogenic role of METTL3 during *in vivo* OS progression.

In terms of how METTL3 is implicated in tumorigenesis, previous studies proposed two distinct m6A-dependent or m6A-independent processes. For instance, Li et al. suggested that the oncogenic effects of METTL3 were promoted by SOX2 mRNA stability in an m6A-IGF2BP2-dependent manner (Li et al., 2019). In other research, METTL3 potentially served as an oncogene in lung cancer by promoting the translation of several mRNAs *via* translation initiation machinery interactions, thus identifying a novel METTL3 mechanism in cancer progression (Lin et al., 2016). In our study, differential DANCR expression between patients was associated with better and worse prognoses; thus, DANCR could be a target of METTL3.

LncRNA DANCR is associated with poor prognoses in a variety of malignant tumors and reportedly has diverse regulatory functions in several cancers, thus functioning as a potential therapeutic target (Thin et al., 2018). We first observed that DANCR expression was associated with a worse OS prognosis. Moreover, similar to METTL3, DANCR silencing also suppressed OS cell proliferation, migration, and invasion. Moreover, silencing both METTL3 and DANCR significantly suppressed OS cell growth and metastasis when compared to the individual silencing of METTL3 or DANCR. Taken together, we hypothesize oncogenic METTL3 was mediated by DANCR activation during OS progression. Given two distinct regulatory processes, we speculated whether METTL3 regulated DANCR expression through m6A modification. From our RIP-qPCR analyses, METTL3 promoted DANCR mRNA stability *via* m6A modification.

In summary, we characterized the oncogenic function of METTL3 during OS progression. METTL3 knockout inhibited OS cell proliferation, migration, and invasion. Moreover, ours was the first study to report that METTL3 promoted OS cell tumorigenesis and progression by increasing DANCR mRNA stability *via* m6A modification.

## Data Availability

The original contributions presented in the study are included in the article/Supplementary Material; further inquiries can be directed to the corresponding authors.
