# Corrigendum: METTL3 contributes to osteosarcoma progression by increasing DANCR mRNA stability via m6A modification

**DOI:** 10.3389/fcell.2023.1167476

**Published:** 2023-07-03

**Authors:** Xinying Zhou, Yang Yang, Yuejun Li, Guojun Liang, Dawei Kang, Bing Zhou, Qingchu Li

**Affiliations:** ^1^ Department of Spine Surgery, Center for Orthopaedic Surgery, The Third Affiliated Hospital of Southern Medical University, Guangzhou, China; ^2^ Department of Orthopedics, Longtan Hospital of Guangxi Autonomous Region, Liuzhou, China

**Keywords:** osteosarcoma, m6A modification, METTL3, lncRNA DANCR, mRNA stability

In the published article, there was an error in Figure 4 as published. The corrected Figure 4 and its caption appear below.

**FIGURE 4:**
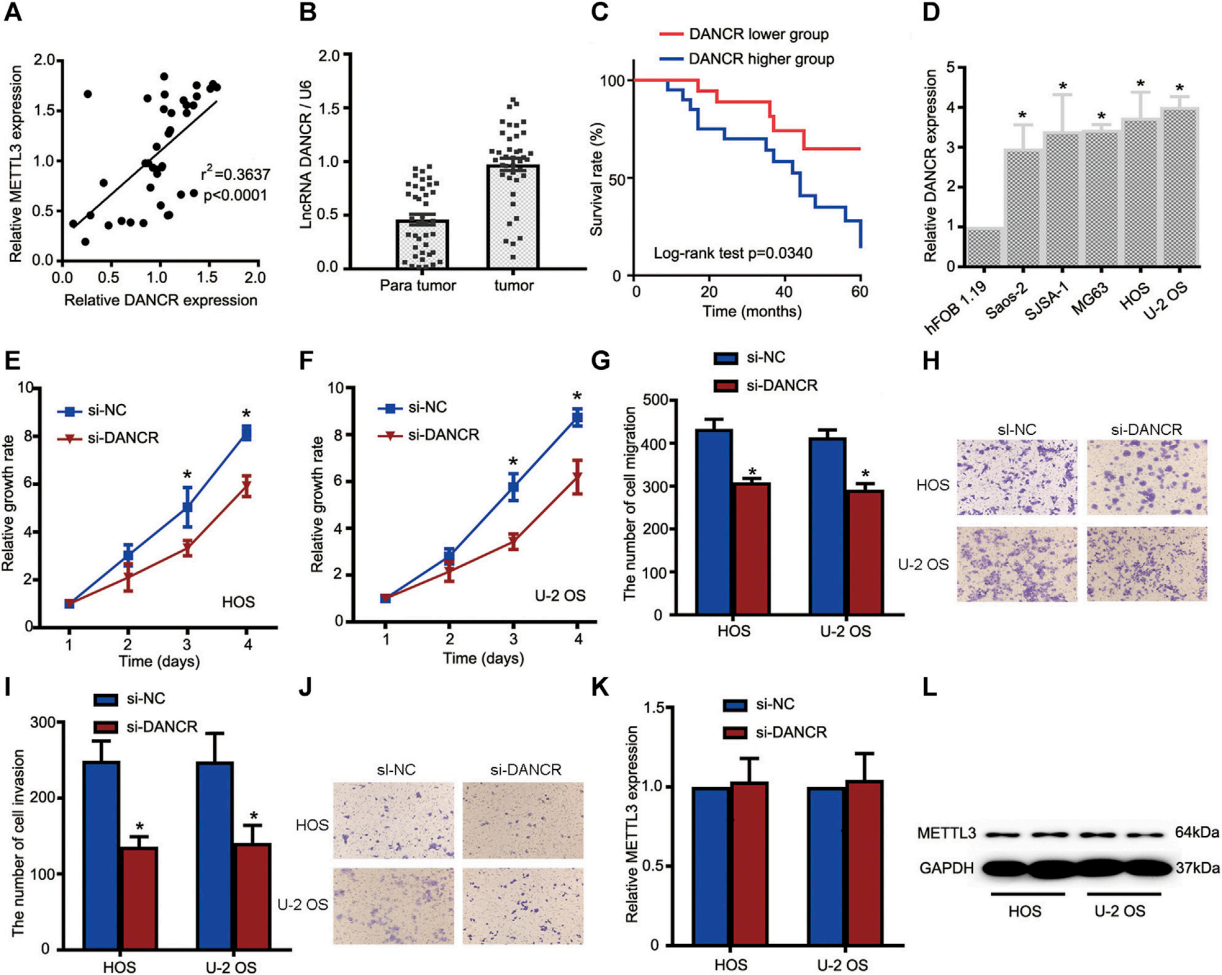
The METTL3/lncRNA DANCR axis promotes OS cell tumorigenesis and progression. **(A)** Correlations between METTL3 and DANCR expression in OS tissue (*n* = 40). **(B)** RT-qPCR showing increased DANCR levels in OS tumor tissues when compared with matched normal tissues. **(C)** Kaplan–Meier survival analyses of patients with OS. Patients with higher DANCR levels showed a relatively poor prognosis. **(D)** RT-qPCR showing elevated DANCR expression in Saos-2, SJSA-1, MG63, HOS, and U-2 OS cells compared with human fetal osteoblastic cells. CCK-8 assays were used to determine viability in HOS **(E)** and U-2 OS **(F)** cells transfected with si-DANCR or the corresponding control. Transwell assays were used to assess the migration activities of HOS and U-2 OS cells. **(G)** Quantitative analyses of migrating cells passing through membranes without Matrigel. **(H)** Representative images of OS cell migration. Transwell assays were used to examine the invasion activities of HOS and U-2 OS cells. **(I)** Quantitative analyses of migrating cells passing through membranes plus Matrigel. **(J)** Representative images of OS cell invasion. **(K, L)** Relative METTL3 expression with or without si-DANCR infected. *p < 0.05.

The authors apologize for this error and state that this does not change the scientific conclusions of the article in any way. The original article has been updated.

