# Supplementary material for: METTL3 Contributes to Osteosarcoma Progression by Increasing DANCR mRNA Stability via m6A Modification
Source: Front Cell Dev Biol. 2022 Jan 12;9:784719. doi: 10.3389/fcell.2021.784719 (PMC8790117; doi:10.3389/fcell.2021.784719)
Supplement: Supplementary file 1 [file Table2.DOCX]

|  | METTL3 low | METTL3 high | p-value |
| --- | --- | --- | --- |
| age | 51.75±12.72 | 54.05±15.71 | >0.05 |
| sex |  |  | >0.05 |
| Male | 10 | 13 |  |
| Female | 10 | 7 |  |
| Histologic response |  |  | >0.05 |
| 2B | 4 | 2 |  |
| 3A | 9 | 8 |  |
| 3B | 6 | 7 |  |
| 4A | 1 | 3 |  |
| METTL3 expression | 0.62±0.24 | 0.75±0.22 | <0.05 |

Table 2. The clinical characteristics analysis from 40 OS patients in this study cohort.
